# Is episodic-like memory *like* episodic memory?

**DOI:** 10.1098/rstb.2023.0397

**Published:** 2024-09-16

**Authors:** James R. Davies, Nicola S. Clayton

**Affiliations:** ^1^ Department of Psychology, University of Cambridge, Cambridge CB2 3EB, UK

**Keywords:** what–where–when, incidental encoding, source memory, mental time travel

## Abstract

Episodic memory involves the conscious recollection of personally experienced events and when absent, results in profound losses to the typical human conscious experience. Over the last 2.5 decades, the debate surrounding whether episodic memory is unique to humans has seen a lot of controversy and accordingly has received significant research attention. Various behavioural paradigms have been developed to test episodic-*like* memory; a term designed to reflect the behavioural characteristics of episodic memory in the absence of evidence for consciously experienced recall. In this review, we first outline the most influential paradigms that have been developed to assess episodic-like memory across a variety of non-human taxa (including mammals, birds and cephalopods), namely the what–where–when memory, incidental encoding and unexpected question, and source memory paradigms. Then, we examine whether various key features of human episodic memory are conceptually represented in episodic-like memory across phylogenetically and neurologically diverse taxa, identifying similarities, differences and gaps in the literature. We conclude that the evidence is mixed, and as episodic memory encompasses a variety of cognitive structures and processes, research on episodic-like memory in non-humans should follow this multifaceted approach and assess evidence across various behavioural paradigms that each target different aspects of human episodic memory.

This article is part of the theme issue ‘Elements of episodic memory: lessons from 40 years of research’.

## 1. Introduction

Mental time travel is the ability to travel through one’s own subjective time, reconstructing memories of past personal experiences, as well as envisioning oneself in possible future scenarios. Within mental time travel, the past, present, and future are deeply intertwined, as our thoughts of the present and future are built upon our memories of the past [1]. This structural foundation of mental time travel, known as episodic memory, is the long-term declarative memory system involving the conscious recollection of personal events [2,3].

Within declarative memory (concerning the encoding and storage of long-term, actively accessible information [2,3]), recalling the past is often differentiated between knowing versus remembering [2], with episodic memory representing the latter. By contrast, semantic memory describes memory for general factual information with, critically, no associated subjective experience during recall (i.e. knowing). This captures episodic memory’s distinctive and defining phenomenology: it involves the conscious experience of ‘mentally reliving’ an event [4]. The distinction is highlighted when examining patients with specific memory function impairments, such as Clive Wearing, who developed a viral infection that effectively destroyed his hippocampus and left him profoundly amnesic [5]. Whilst his semantic memory remains largely unaffected, his ability to engage in episodic memory is entirely diminished. Wearing is able to communicate effectively with others, play the organ and conduct a choir, but rapidly forgets events he experienced only seconds before. This separation is true of other amnesic patients, especially those resulting from hippocampal damage, whereby semantic memory largely remains intact but severe deficits in episodic memory are observed [6–9]. Similarly, episodic memory, relative to semantic memory, declines rapidly and dramatically with normal ageing [10–15].

In addition to his inability to remember his past, Wearing is also unable to imagine his future [5]. Again, this shared deficit is true of other amnesic patients with comparable hippocampal damage [16–20], and thus the hippocampus seems critical in both the retrieval of episodic memories and the generation of imagined future events. Indeed, functional magnetic resonance imaging studies on healthy subjects reveal that the same brain activation patterns are generated when people imagine the future as when they remember the past [21–24], suggesting that both processes depend on a common neural substrate [17,25–27].

While the term ‘memory’ is often associated with analogies such as a library, implying that it is a fixed bank of information, this is more akin to semantic memory than episodic recollection [28]. By contrast, the retrieval of episodic memories reconstructs the memory, often at the expense of accuracy [29,30]. The event fragments, encoded automatically over a single exposure [31], are reassembled and interwoven with semantic information to ‘recreate’ the narrative of the memory, rather than accurately replaying a copy of the event [32].

While episodic memory does have its own distinct features, i.e. memory for personal events that are mentally re-experienced, and appears to be centralized in the hippocampus, it is not a completely independent system and is influenced by a number of wider cognitive processes and cortical areas. For instance, the medial temporal, medial prefrontal, midline and parietal brain regions make up a ‘core network’ that is engaged during episodic recall and imagining the future [25–27,33–36], and functionality in brain areas involved in visual perception [36,37] and visual imagery [38–41] have been linked to the experienced vividness and visual perspective during episodic memory recall [42]. As such, emotion- and sensory-related brain regions are recruited during remembering and contribute to the phenomenological features of episodic memory recall [37]. Furthermore, although episodic and semantic memory are considered to be distinct, the episodic components encoded in memories for certain events, say a trip to the shops, are scaffolded onto a semantic structure representing general information on how such events typically exist and transpire [43–45], i.e. the exchange of money for goods within a designated building. Consequently, episodic memory can be conceptually broken down into its various supporting cognitive processes (that are crucial, but not exclusive, to episodic memory), which combine to generate the ostensibly distinct memory system introspectively known to us.

## 2. Paradigms used to test episodic-like memory in non-human animals

While we use episodic memory in all aspects of life, allowing us to capture and recall a wide array of experiences from remembering what we ate for breakfast to how we felt after the announcement of Brexit, it is thought by many psychologists to be a uniquely human ability (e.g. [5,28,46–48], but see [49,50]). They argue that while animals have a sophisticated and sensitive semantic knowledge of their environment, they cannot consciously recall and relive specific past experiences as we can [46]. However, as evidence for conscious episodic recall in humans is centred around language-based reports, and with there being no widely accepted non-linguistic markers of consciousness [51], it is potentially impossible to establish if non-human animals have true, conscious episodic memory. In light of these issues, researchers have developed multiple behavioural paradigms to investigate aspects of non-human animals’ memory recall, using the subjects’ behaviour in tightly controlled tasks to infer their underlying mental processes. Without evidence of conscious recall, however, this ability is termed ‘episodic-like memory’ [52]. Accordingly, for the purposes of this review, we define episodic memory as the memory system involved in the long-term conscious recall of personal experiences, along with its associated phenomenological components (e.g. emotion and visual imagery). However, we use the term episodic-like memory to describe a memory system involved in the long-term recall of personal experiences as assessed through behavioural characteristics, i.e. when we do not have access to the subjects’ subjective, phenomenological experience during recall. As such, while the two terms are not necessarily mutually exclusive (as episodic-like memory may be assessed in human subjects with full episodic memory, in which case it simply refers to the method of assessment), and episodic-like memory may present differently across non-human taxa, we hereafter refer to event memory recall in humans as episodic memory, and the equivelant memory recall in non-human animals as episodic-like memory. While this distinction may simply represent limitations in the data available for measurement (i.e. verbal report versus inferences from behaviour), there may also be fundamental differences between the two memory systems (and indeed variation within episodic-like memory between species), as episodic memory is influenced by a variety of wider brain structures and cognitive abilities that certain non-human species may or may not possess. Consequently, episodic-like memory systems may be limited in form and function compared with full episodic memory (e.g. it may be relatively restricted to the hippocampus, resulting in diminished experiential richness [53]), perhaps representing an evolutionary precursor ability [54], or alternatively, an evolutionary convergence for remembering personal events [55,56].

### (a) What–where–when memory

Using Tulving’s original definition of episodic memory, which states that it ‘receives and stores information about temporally dated episodes or events and temporal-spatial relations among these events’ [2, p. 385], Clayton *et al*. [57] argue that the simultaneous retrieval and integration of information about the ‘what’ and ‘when’ of unique experiences (‘temporally dated experiences’), and ‘where’ they occurred (‘temporal-spatial relations’) can be behaviourally demonstrated in animals. As such, three criteria were proposed for a demonstration of episodic-like memory via the what–where–when memory paradigm [58]. These criteria are the *content*: recall of the ‘what’, ‘where’ and ‘when’ information associated with an event; the *structure*: the what–where–when features are fully integrated and form a single representation; and *flexibility*: the what–where–when information is encoded in a way that does not specify its use in subsequent behaviour.

Coining the term episodic-like memory, Clayton & Dickinson [52] demonstrated that scrub-jays (a member of the corvid family) can encode and use what–where–when memories. In the wild, scrub-jays cache both perishable foods (e.g. insect larvae) and non-perishable foods (e.g. nuts) for consumption at a later date. By using this natural phenomenon with food items that degrade at differential rates, these studies demonstrated that these jays remember the ‘what’ (food type), ‘where’ (cache location) and ‘when’ (time of caching relative to retrieval) *content* of trial unique caches they made when recovering them after varying time intervals [52,59]. Furthermore, they show the correct search behaviour even when the event memories share the same ‘what’ and ‘where’ information, but differ with respect to ‘when’, demonstrating that these memories are formed in an integrated *structure* [59], and are able to integrate novel semantic information (acquired after the event) with the what–where–when event information, allowing for cognitive *flexibility* [60].

While many researchers have since conducted studies on a large variety of species using the what–where–when paradigm, including other birds [61,62], rodents [63–74], primates [75,76], dogs [77] and cephalopods [56,78], these predominantly focus on the *content* criterion, and as a result, there is little evidence for the *structure* (but see [68,75]) and *flexibility* (but see [74]) criteria in the literature.

### (b) Incidental encoding and unexpected question

That said, training animals to explicitly encode and report information in an anticipated memory test, as is required for what–where–when memory experiments, may result in the application of non-episodic mechanisms to solve these tasks [74,79–84]. If, at the time of encoding, an animal can anticipate that the presented information is relevant for an upcoming memory test, then they could preselect a future action using a non-episodic memory trace [85–88] and subsequently use this action without actually *remembering* back to the encoding event *upon* presentation of the memory assessment (the central hypothesis of episodic(-like) memory [85,89]). By contrast, when information is encoded incidentally and subsequently tested in an *unexpected* memory assessment, the transformation of this information into a preselected future action is impossible [79,82,85,90], as you cannot predict the importance of certain information and use this to plan future behaviour when you have no reason to expect a memory test for this information [85,86].

This situation describes an alternative methodology to test episodic-like memory in non-human animals: the incidental encoding and unexpected question paradigm [79]. The basis for this paradigm lies in the automatic encoding characteristic of human episodic memory, i.e. that when we participate in episodic memory and recollect a particular experience, we may retain details from that event which held little significance to our needs, thoughts or desires at that time. Despite not being marked as important enough to explicitly encode into memory, this ‘incidental’ information is automatically encoded within the memory [91] and is later retrieved as part of a holistic representation of the event [92]. A key characteristic of this type of remembering is that we may not know that certain information surrounding the event would later be called upon to be remembered, but we are able to do so anyway [86]. Consequently, this paradigm seeks to behaviourally represent the incidental encoding feature observed in human episodic memory by prompting subjects to unexpectedly recall incidentally encoded details about a specific event.

Since its emergence [79], researchers have used the incidental encoding and unexpected question paradigm to explore episodic-like memory in various non-human animals, including pigeons [79–81], rats [74,82,93,94], dogs [95–97], cats [98], corvids [92] and dolphins [83]. These studies demonstrate that multiple non-human species are able to encode and recall incidental information within remembered events, such as spatial locations [80,81,83,93,97,98], visual markers [92], the identity of experimenters [83], the presence (or absence) of food [74,82], odour cues [94], as well as aspects of their own [79–81,93,95] and other’s behaviour [96]. By focussing on a separate aspect of human episodic memory to the what–where–when memory paradigm, these studies develop an alternative methodology to investigate episodic-like memory and thus have the potential to strengthen previous findings (e.g. [64,74,82]).

### (c) Source memory

Another promising method to investigate episodic-like memory in non-human animals is to test for the encoding and recall of source memory [99]. When we episodically recall an experience, we are able to retrieve certain features, including the specific temporal, contextual, perceptual, and affective details, that are tied to these memories [100]. To recall these encoded details, we must engage in episodic memory to mentally travel backwards in time to re-experience the content of our personal memories and access these features through source memory processes [101]. Source memory is a representation of the origin of the previously acquired information, and is retrieved through monitoring these features [99]. In other words, source memory is a memory for the context in which a memory was formed. Monitoring the source information of memory allows us to differentiate between two similar remembered events [100,102], and as source memories are embedded within episodic memories, they are a characteristic component of episodic memory in humans [99,101,103,104].

Studies investigating source memory in non-humans are relatively scarce [68,99,101,103,105]. That said, Rhesus macaques have demonstrated evidence for source memory formation in an item versus source memory discrimination task, in which subjects were asked to recall the items they previously encountered, discriminating between these and novel items, and then identifying the context surrounding their presentation [103]. Furthermore, rats were shown to be able to discriminate between self- or experimenter-generated information [99]; a form of source memory known as reality monitoring [100]. In the latest source memory experiment with non-human animals, cuttlefish demonstrated their ability to differentiate the sensory modality (smell versus sight) by which they acquired foraging information [101].

Again, this methodology targets a different characteristic of human episodic memory from the previous paradigms. Therefore, while the source memory literature in non-humans is currently largely lacking, this paradigm, with variations representing different modalities of source memory, shows great potential for investigations into episodic-like memory, again building on previous findings (e.g. [99,105]).

## 3. Is episodic-like memory *like* episodic memory?

Decades of research have built a large body of knowledge on the episodic-like memory abilities in many non-human animal species. However, although humans appear to use conscious episodic memory in episodic-like memory tasks in some instances [106], in others they fail the tasks altogether [107,108]. Furthermore, human performance across different paradigms is not necessarily related [109], suggesting that distinct psychological processes may be employed for each [107,109,110]. We now examine whether various key features of episodic memory are represented in episodic-like memory, identifying similarities, differences, and gaps in the literature.

### (a) Is episodic-like memory dependent on the hippocampus?

As discussed, episodic memory in humans is dependent on a functioning hippocampus [46]. Does the same structural dependence exist for episodic-like memory in non-humans? Indeed, many studies use animal models to understand the human brain (e.g. [34,68,88,94,99,111]), assuming some level of continuity in form and function. Although often not using conventional episodic-like memory paradigms, research in these studies shows that damage to the hippocampus is associated with defects in episodic-related memory functions in monkeys [112] and rodents [66,113,114]. These observations are supported by research using established episodic-like memory paradigms, showing that rodents with hippocampal lesions fail in both what–where–when tasks [70,72] as well as incidental encoding tasks [82].

Furthermore, specific cells within the mammalian hippocampus, known as ‘place cells’ [115], preferentially fire when an animal is in a particular spatial location, and thus as they move through a series of locations, an ensemble of cells fire in corresponding sequences. These cells also activate during ‘replay’ events, known as ‘sharp-wave-ripples’, during periods of rest which are thought to be involved in learning and memory in rodents [116] and have been linked to episodic memory recall in humans [117]. By observing the sequential firing of these place cells, researchers have shown that the rat hippocampus consistently engages in retrieving previous experiences during pauses in waking behaviours [118], for example when stopped at a decision point in a maze [116,119,120]. As well as encoding spatial information about past, present, future and distant locations [119,121–123], evidence suggests that place cell activity in rats can also encode information about the ‘what’ (e.g. odours and their status, i.e. match/non-match [124–126]) and ‘when’ (i.e. elapsed time [127,128]) of events. In fact, the sequential activation of place cells is thought to reflect specific episodic-like memories, occurring across similar time scales to the actual experience, all while the rat is concurrently engaged in other activities (and thus is not confusing imagined representations with current reality) [127].

It seems, therefore, in mammals at least, that an unimpaired hippocampus is required for a functioning episodic-like memory system. How then, does episodic-like memory function in animals with distinct neurophysiological structures, such as birds and cephalopods? Research on humans, non-human primates and rodents suggests that the connectivity between the hippocampus and the neocortex in mammalian brains allows for close interactions and information pathways between these structures [33,129]. During the encoding and retrieval of information contributing to episodic(-like) memories, the hippocampus employs distinct neurophysiological rhythms to synchronize hippocampal and neocortical activity, with the direct connections from the hippocampus to the prefrontal cortex appearing particularly important. While extensive evidence in birds suggests that the processing and storage of spatial information contributing to episodic-like memory is dependent on the avian hippocampus [130–132], evolutionary divergence in pallial organization and neurophysiology (the most representative pallial structure in mammals is the neocortex, but is the dorsal ventricular ridge in birds [133]) indicates that there may be fundamental differences in how these structures process information [53,134]. Indeed, it appears that, in contrast to the mammalian hippocampus, the avian hippocampus does not receive a comparable input from the high-order multimodal associational areas, suggesting that the information contributing to episodic-like memory in birds is more limited and is processed in a different manner than compared with mammals [53]. This dissimilarity is likely to be even more extreme in species that are even further phylogenetically and neurophysiologically separated, such as cuttlefish which do not even possess a hippocampus [135]. Instead, the structure representing the epicentre for learning and memory in cuttlefish is known as the vertical lobe, which presents similarities to vertebrate hippocampus formation in its connectivity and functionality [135–137].

While it seems that mammalian and avian episodic-like memory does, like human episodic memory, depend on a functioning hippocampus, the way in which inputs from other brain regions interact with the hippocampus to form episodic-like memories may be fundamentally distinct. As such, the anatomical differences, and the resulting neurophysiological differences, may mean that the processes of memory being assessed in episodic-like memory tasks across these taxa may not be directly comparable. This inference is more extreme with species who do not even possess a hippocampus, such as the cephalopods, but instead have an analogous structure involved in memory, leading to further neurophysiological divergence.

### (b) Does episodic-like memory show age-related decay?

In humans, episodic memory is well known to decline with normal ageing [10,11] and deteriorates the earliest and most dramaticlly compared to other forms of memory [12–15]. Age-related changes to the hippocampus are generally similar across mammals [138], and research on memory in rodents and monkeys, although again not strictly in line with episodic-like memory criteria, suggests that both spatial (what–where) [139–142] and temporal (what–when) [142,143] elements of memory decline alongside typical age-related changes within the brain.

Using a variation of the what–where–when paradigm (what–where–*which*), aged mice were observed to perform with less success in comparison to their younger counterparts [144,145]. Interestingly, this effect was not apparent in the standard what–where–when task, leading the authors to conclude that non-episodic solutions are possible in what–where–when tasks, as suggested by others [79,85]. Furthermore, older dogs and apes who were otherwise healthy showed lower performances in an incidental encoding and what–where–when test, respectively, compared with younger conspecifics [75,146]. These findings echo those found in human episodic memory research on ageing (e.g. [11]). While the evidence suggests that we may be able to assume that animals with similar brain structures are susceptible to similar effects of physiological ageing in regards to episodic(-like) memory performance [78], as before this leads to the question of whether the same effects are observed in animals further phylogenetically separated with somewhat different brain structures, such as birds, or even significantly different, such as cephalopods. While we know of no study directly assessing the development of episodic-like memory in birds (but see [147,148]), they do share a similar hippocampal structure to mammals, albeit without the connections and influences of other structures in generating episodic-like memories [53], as discussed previously. However, in a what–where–when study the performance at test of aged-adult cuttlefish did not differ from sub-adult cuttlefish [78] (but interestingly did reach learning criterion faster), revealing that the observed age-related decline in episodic(-like) memory may involve the hippocampus.

### (c) Is episodic-like memory a reconstructive process?

Episodic memory in humans is reconstructive in nature, in that the features of these memories are separated and stored individually at encoding, and then recombined at retrieval to recreate the event narrative [30]. When events are reconstructed during recall, the associated features are bound with the memory to form a single representation of the event. Episodic memory reconstruction is facilitated through source monitoring processes, which allows two different episodes that share multiple common features, such as occurring in the same place with the same person, to be differentiated between [100]. Therefore, in order for the reconstruction process to be successful, multiple features would need to be retrieved and assessed in order to allow the discrimination between these two similar memories.

Following these observations, two lines of evidence can be drawn to support the notion that episodic-like memory is, or at least has the potential to be, a reconstructive process. First, as the reconstruction and binding of event features rely on source monitoring, evidence of source memory in non-human animals would suggest that these information-monitoring processes exist. Without memories for source information associated with an event memory, the retrieval and assessment of the memory features during reconstruction would not be possible. Therefore, studies on non-human source memory [68,99,101,103,105] would satisfy this prerequisite. Second, studies showing that episodic-like memories are bound with multiple details surrounding the event, therefore providing distinguishing features, would allow for event reconstruction. Indeed, a failure to bind multiple features of an episode has been theorized as the cause of human participants’ failure in a what–where–when task [107]. Having demonstrated that jays could differentiate between two similar events [59], this suggests that the ‘when’ information was bound to each memory during reconstruction which, facilitated by source monitoring processes, allowed them to alter their decision-making in accordance with each specific caching event. The same binding of multiple event features, permitting the discrimination between two similar events, has also been demonstrated in rats and apes [68,75]. Consequently, evidence through feature binding suggests that the two most published animal models for episodic-like memory (jays and rats) have the potential to reconstruct their episodic-like memories at recall.

In addition to successes in memory tests, evidence may also come from when memory fails. As inaccuracies in episodic memory are often related to reconstruction [29,30], similar memory failures in episodic-like memory may reveal their underlying processes. For example, Rhesus macaques showed a decline in source memory discrimination performance (but not item memory) as the retention interval increased between encoding and retrieval [103]. This pattern is typical of human episodic memory, both in real-life episodic remembering as well as that observed experimentally, with performance in tasks deteriorating with increases in the retention interval [149,150], and is observed in other episodic-like memory studies [96,101,151].

Furthermore, as event fragments are reconstructed and interwoven with semantic information to reform episodic memories [29,30], the ability to integrate semantic information with episodic-like memories would support the evidence outlined above. Scrub-jays were observed to integrate novel semantic information with an original episodic-like memory of a caching event, and importantly, acquired this information after said event [60]. This suggests that at the time of cache retrieval, the episodic-like memory was recombined and integrated with (novel) semantic information, providing further evidence of reconstruction in episodic-like memory.

### (d) Are episodic-like memories automatically encoded over a single episode?

Episodic memories are rapidly acquired over a single learning episode, without the need for subsequent rehearsal [31]. Indeed, memories that require rehearsal over numerous exposures are thought to represent fundamentally distinct memory processes to episodic memory and to have evolved to serve separate psychological functions [152]. Furthermore, event details are often encoded automatically, in that we often do not consciously attempt to memorize these details at the time [31]. In contrast to the encoding processes in these other forms of memory, the encoding of episodic memories may require specialized neural mechanisms that are distinct from those needed in perception and attention [153], and thus information is encoded automatically whether or not the details are attended to [154].

An essential feature of experimental design required in any episodic-like memory paradigm is that the encoding of the event in question occurs over a single unique experience [52,58]. Otherwise, non-episodic mechanisms can be used to solve memory tasks after repeated training [155,156]. Consequently, these studies by design show that episodic-like memories are formed over a single episode. Furthermore, the incidental encoding and unexpected question paradigm, also by design, directly represents the automatic encoding feature of episodic memory [79]. Therefore, these studies provide evidence that episodic-like memory, if determined via incidental encoding, involves automatic encoding. Experiments using other paradigms, however, typically involve repeated trials to train the subjects to attend to certain information such as perishability rates of food types, resulting in the explicit encoding of these details. Subsequently, these other studies cannot be used to answer this particular question about episodic-like memory.

Furthermore, when rats received a singular experience of a mild shock upon entering a ‘shock-zone’ in a track, the place cells representing this zone were activated when the rats merely approached it, causing them to avoid the zone [157]. This suggests that when showing the avoidance behaviour, the rat is representing the likely occurrence of an event associated with the context of a specific spatial location (the shock), critically based on only a single experience.

### (e) Does episodic-like memory correlate with an ability for future planning?

In humans, amnesic patients who cannot remember their past also cannot think about their future. Does the same association exist with episodic-like memory? Problematically, however, while behavioural paradigms have also been developed to assess future planning in animals, there are abundant disagreements regarding the interpretation of the results in these experiments. As a full discussion of these issues will detract from the main focus of this review, these concerns will be only briefly mentioned here.

In caching experiments, scrub-jays [158], but not Canada jays [159], have been shown to plan for the future by preferentially caching in places where they have learned to expect not to receive food the following morning. Furthermore, both scrub-jays and Eurasian jays appear to plan for future needs that differ from their current motivational states [160,161], which is clear evidence against the ‘Bischof-Köhler hypothesis’, stating that only humans can override their current desires (e.g. hunger) in order to satisfy possible future desires [28]. While rats [162] and Rhesus macaques [163] appear to fail at this task, other monkeys (squirrel monkeys) were successful [162]. That said, both this and the previous paradigm have been criticized for not showing evidence of true foresight as seen in humans [164,165], or have failed to replicate [166]. However, in a preregistered ‘spoon test’ [4] experiment, designed by leading researchers on opposing sides of the debate regarding the human uniqueness of mental time travel (namely, Clayton and Suddendorf), New Caledonian crows were demonstrated to retain representations of various possible future scenarios, derived from previously acquired information, and then perform specific actions at present to plan for different possibilities [50]. In similar experiments, apes also appear to plan for future possibilities by choosing and retaining appropriate tools for future use [167,168]. However, these studies were importantly conducted without adequate controls to rule out simple associative explanations [169], as were implemented in the New Caledonian crow study ([50]; but see [170]). Furthermore, although not adhering to any generally approved future planning paradigm, cuttlefish have also been demonstrated to show flexible and future-dependent foraging decisions in response to dynamic prey conditions [171]. Similarly, and in contrast to the previous study [162], rats have been argued to show behavioural and neurological evidence for what appears to be future planning [172], or at least, the precursors to planning [89,173,174]. For instance, reactivation of place cells relating to the shock zone experience [157] occurred in a sequence representing the path from the rat’s current position to the shock zone, raising the possibility that replay allows for future planning. As such, pathways to remembered goals were ‘pre-played’ before they engaged in goal-directed navigation through an area, even when the specific routes were novel [172]. This pre-play directly predicts future navigation behaviour, suggesting its role in future planning. While no experimental research exists on the future planning abilities of dolphins, an observational study with Risso’s dolphin suggests that they use information about prey learned throughout previous dives to plan foraging choices in the next dive [175].

Overall, while the evidence for future planning in non-human animals remains highly controversial, the strongest evidence that exists currently [50,164] comes from corvids who were also the original animal models for episodic-like memory [52] (although these abilities may vary within this taxa [158,159]).

### (f) Is episodic-like memory flexible across contexts?

The generalizability of human episodic memory is one of its most apparent and important features. This then raises the question of whether episodic-like memory is flexible across contexts, or alternatively, a highly specialized adaptation and restricted to certain natural behaviours.

Much of the evidence for episodic-like memory in birds, particularly using the what–where–when paradigm, has revolved around food caching in species that are highly specialized in this behaviour, namely scrub-jays [52,59,60], Eurasian jays [92], magpies [61] and black-capped chickadees [62]. While some of these experiments required the birds to remember details of caches that they did not make themselves [62,92], this is still under the remit of their naturally occurring behavioural repertoire. Indeed, chickadees failed to recall ‘when’ information when tested in non-natural settings [62]. Similarly, rodent studies on episodic-like memory typically involve foraging for food within mazes, akin to tunnels, and so also centre around natural (food-related) contexts. Therefore, it is difficult to draw any conclusion from these studies of whether episodic-like memory is restricted to evolved behaviours or whether it is analogous to the generalizability observed in human episodic memory [48]. Some studies, however, use non-natural methodological elements to test for episodic-like memory [75], such as pecking or approaching certain shapes or coloured panels that are presented in front of the subjects [56,78–81], or requiring subjects to recall their own or others’ previous actions out of an array of behaviours [95,96]. That said, although these methodologies are not based on highly specialized behaviours, they do require repeated training to associate certain information, decisions, and actions with food rewards, and then test for memories based on another unique, but critically similar, episode. As such, although the recalled information is not necessarily associated with the species’ natural behaviours, they do not occur spontaneously without training to attend to them. However, dolphins have been demonstrated to be able to spontaneously recall information that was not trained to be attended to, representing both spatial and social information [83]. The recall of incidental social information is particularly interesting, as this concerned human experimenters and was encoded visually, which does not represent cetaceans’ natural social information and primary method of communication (auditory). Subsequently, it could be argued that they were able to generalize social information across species, demonstrating some level of episodic-like memory flexibility, although it is likely that the intensive training they received over their lifetime facilitated this flexibility [176].

That said, as humans we too receive what can be thought of as ‘training’, as we learn (often socially) to form various associations and to attend to certain informational variables. Furthermore, it could be argued that the environment we have built around us is essentially our natural habitat, as although civilization and the resulting modern ways of life were not present during the majority of our evolutionary history, much of the socio-ecological practices we engage in (i.e. our behavioural repertoire) remained consistent throughout (albeit within different physical and social structures). For instance, it is highly likely that we have evolved to satisfy the cognitive demands of language, tool use, cooperation, and complex sociality that make up the foundations of our lives both in prehistory and today and currently still constitute the base components of our episodic memories (e.g. recalling how you put your phone back together or remembering a friends birthday party). Nevertheless, we are still able to generalize our episodic memory system across varied socio-ecological aspects of our lives to an ostensibly greater extent than observed in non-human episodic-like memory, which for the most part is restricted to food-acquiring behaviour. However, our understanding of the evolution of episodic(-like) memory would benefit greatly from research that considers the extent of this contextual flexibility in humans.

While there is little evidence to suggest that episodic-like memory is akin to the domain-generality of human episodic memory (even within our evolved socio-ecological parameters), this may simply represent a dearth of relevant research, as exploiting natural behaviours is a clever and efficient technique to explore the cognition of animal subjects. Alternatively, however, this may reflect a large distinction between episodic memory and episodic-like memory.

### (g) Does episodic-like memory involve a subjective experience?

Whether or not episodic-like memory recall is accompanied by some form of subjective, conscious experience, is the conceptual foundation behind the term episodic-like memory. While the what–where–when memory paradigm was originally designed to bypass this element of human episodic memory, aspects of other episodic-like memory paradigms may shed some light on the contents of an animal’s consciousness during recall.

To access incidentally encoded information, an animal must recall a holistic representation of the episode, containing many details surrounding the event as well as the event narrative sequence, and subsequently replay, target and manipulate this information within the event representation [85,92,94,114]. Indeed, recent evidence demonstrates that humans have conscious access to incidentally encoded information within memories and can target this information in order to solve memory tasks [154,177–179]. While similar research suggests that other memory processes we introspectively believe to involve conscious awareness (i.e. working memory) may also exist in the absence of conscious experience [179–181], these observations nevertheless raise the possibility that non-human animals may also have conscious access to incidental information within memories. In addition, as episodic memories are based on past perception, success in source memory tests depends on the subjects’ capacity to retrieve what was perceived, as well as how it was perceived. This retrieval relies on a personal assessment of the subject’s own previous internal experiences [101]. As such, while what–where–when memories represent external information (i.e. ‘what happened, where, and how long ago’ and/or ‘what happened where and in which context’ [182]), source memories, regarding the perceptive signal responsible for the generation of episodic-like memories, are based off internal information representing past senses (i.e. ‘I saw this happen’ versus ‘I heard this happen’). Therefore, this paradigm may provide more detail about the subjective experience of the animal during recall (i.e. ‘what did I just experience?’) [101]. However, it must be noted that even in humans the connection between source memory for perceptual details and conscious recall (i.e. ‘remember’ judgements) is not necessarily universal and clear-cut [183].

Neurological evidence may also shed light on this question. As previously discussed, research on place cells has shown that the rat hippocampus consistently engages in retrieving previous experiences during pauses in waking behaviours [116,118–120]. While retrieval is conceptually distinct from the conscious experience of recollection [184], for which retrieval is essential but not sufficient, recent remodelling of sharp-wave-ripple events suggests that this neuronal replay often occurs at ‘real-world’ speeds that reflect actual experience [185], as observed in other studies [127]. Furthermore, this reply often occurs during sleep and as activity takes place within the visual cortex as well as the hippocampus, it may directly relate to an experience of perceptual imagery during sleep states [186]. However, although hippocampal sharp-wave-ripples have been linked to visual episodic memory recall in humans [117], they do not necessarily represent the neural markers of subjective experience in non-human animals.

Nevertheless, these observations highlight that research on other comparable mental processes may suggest that non-human animals have the capability to undergo subjective, conscious experiences. As such, behavioural and neurological research on dreaming in non-humans raises the possibility that animals are capable of consciously experiencing internally generated mental representations in the form of dreams [116,187–193]. In humans, recent evidence shows that mental time travel and dreaming may be generated through similar networks in the brain [194,195], share parallels in their (re)constructive processes [196–199] as well as deficits owing to injury and disease [200,201]. Furthermore, while the replay of complete episodic memories is rare during dreaming [197], dream imagery and content is made up of fragmentations and transformations of episodic stimuli [196–198]. This suggests that rather than dreaming being a completely distinct phenomenon to mental time travel, they are both part of a continuum of spontaneous, offline (re)constructive thought, generated across sleep and waking states [199,202]. While research on animal dreams is similarly controversial, as neuronal replay during sleep may simply function in memory consolidation [116], these observations demonstrate the use in assessing converging lines of evidence when attempting to investigate complex cognitive phenomena, such as conscious experience.

Furthermore, as consciousness is multidimensional with many different and complex parameters [203], some of these features, but not others, may be present in episodic-like memory recall (the combination of which depending on the species in question [204]). This is particularly pertinent when focussing on the anatomical and neurophysiological distinctions between different taxa. For example, as the avian hippocampus, in contrast to the mammalian hippocampus, does not receive comparable input from high-order cortical areas, components of daily experiences (processed in these areas) may not contribute to episodic-like memory formation and retrieval [53]. As such, the *experience* of episodic-like memory may differ across taxa. Consequently, while we are still currently bound to the term episodic-like memory, as the puzzle of conscious recall remains, we believe that further empirical investigation (as opposed to simply bypassing the question) may lead to tangible answers relating to the subjective experience of episodic-like recall.

## 4. Conclusions and future directions

In summary, since the conception of the term episodic-like memory, major research effort has enriched our understanding into the ability of non-human animals to recall the past, namely through the what–where–when, incidental encoding, and source memory paradigms. These investigations have been spread across phylogeny, shedding light on the memory abilities of a variety of taxa from rodents and primates to birds and cephalopods.

While, by design, the term was intended to bypass the defining phenomenology of human episodic memory, we have reviewed and highlighted various other features of episodic-like memory that do appear to be shared with episodic memory, as well as those that may reflect anatomical, mechanistic, functional and/or developmental distinctions between these memory systems. We conclude that there is sufficient evidence to claim that, like episodic memories, episodic-like memories are formed over a signally occurring episode, and, in mammals at least, are dependent on the hippocampus while exhibiting a natural age-related decay in performance. Furthermore, albeit with less certainty, that there is at least some evidence to suggest that both processes are reconstructive in nature, involve the automatic encoding of associated event details, and correlate with the ability to plan for the future. Similarly, while still lacking in concrete evidence, aspects of the episodic-like memory literature (as well as data regarding similar processes) raise the possibility that animals can undergo some degree of subjective experience during recall, although not necessarily as we would recognize it. By contrast, there is relatively strong evidence to suggest that in non-mammalian taxa, namely cuttlefish and birds, episodic-like memory can exist in the absence of a hippocampus, or in the absence of high-order pallial inputs into the hippocampus, respectively, with these anatomical differences potentially resulting in drastic consequences regarding the structure, development, mechanisms, and experience involved in episodic-like memory across these species. Furthermore, although likely in part owing to a scarcity in related research, there is little evidence to suggest that episodic-like memory is flexible across contexts in the same was as human episodic memory. As this feature represents one of its most significant aptitudes, this distinction may reflect dramatic limitations relating to the use and evolution of episodic-like memory in non-human species. Therefore, future investigations into how episodic-like memory is presented in different phylogenetically, ecologically and neurologically distinct cognitive systems [204], as well as its contextual limitations (and the limitations of human episodic memory), hold particular importance. Furthermore, the field will be dramatically enhanced by more detailed investigations into the neurological underpinnings of episodic(-like) memory (e.g. place cells and sharp-wave-ripples) across different taxa. While this research is mostly limited to species easily amenable to neurological-based experiments (e.g. rodents), assessing neurophysiologically distinct groups, such as birds and cuttlefish, will be particularly informative as our understanding is largely limited to mammalian brains.

In addition to these research avenues, we argue for the use of an ‘episodic-like memory test battery’, with a combination of what–where–when (including *structure* and *flexibility* criteria), incidental encoding, and source memory tests. As in all the available behavioural paradigms evidence of episodic-like memory is based on the exclusion of alternative, non-episodic test solutions, it is unlikely that any single methodology will provide an exhaustive conclusion [85]. If a species’ performance on tests across multiple paradigms, which often use considerably different techniques, are each suggestive of episodic-like memory, it is unlikely that all of these tests have failed in the same way to all lead to the false appearance of episodic-like memory. As such, the combination of multiple, converging lines of evidence using different approaches will lead to much stronger inferences [111]. Furthermore, human episodic memory encompasses a variety of cognitive structures and processes amalgamating into an episodic memory system, and importantly each episodic-like memory paradigm targets a different aspect of this system [85,109]. For example, what–where–when memory tasks isolate the individual *components* of human episodic memory (factual, spatial and temporal), assess if an animal can recall information that is flexible usage depends on the passage of time, and whether this information is declarative in nature. However, the incidental encoding and source memory paradigms assess whether non-humans are able to recall information that can only have been encoded in certain contexts (incidental information and source information, respectively) using *processes* associated with episodic memory in humans (automatic encoding and source monitoring, respectively). Therefore, to provide comprehensive evidence of episodic-like memory in any given species, as well as to accurately model all the different aspects of human episodic memory, researchers should combine results from varying paradigms and look for converging lines of evidence.

Lastly, as studies on animal cognition in captivity with human raised subjects cannot accurately replicate the conditions under which the cognitive ability in question evolved, efforts should be made to assess episodic-like memory in wild animals in their natural habitat [205]. Indeed, many natural behaviours observed in the wild may suggest an episodic-like memory ability and consequently represent valuable opportunities for use in experimental research. For example, observational data on wild grey-cheeked mangabey foraging behaviour may suggest a form of memory that includes recalling the ‘what’ (fruit), ‘where’ (tree location) and ‘when’ (timing relative to fruiting) aspects of previous foraging experiences [206], while incorporating recent weather conditions (semantic information) with these memories to flexibly dictate decision-making [207]. Similarly, Sumatran orangutan long calls, in which flanged males emit loud calls to convey social information to females and other males, predict their future travel trajectories [208], perhaps involving remembered past travel routes and outcomes. While these behaviours represent promising candidates for the study of episodic-like memory, the interpretations that can be reasonably drawn from these data would be significantly strengthened through controlled experimental research, as, for example, it is often impossible to rule out the influence of various environmental cues in dictating behaviour [208]. As such, observations alone are not sufficient to conclusively identify underlying cognitive mechanisms [209]. While some researchers have attempted to experimentally investigate aspects of episodic-like memory in natural settings [210], we know of no published experimental studies assessing wild subjects on their memories for personal, unique events, that cannot be explained through non-episodic-like mechanisms. This represents an important gap in the literature and addressing this gap would lead to more astute investigations of the evolution of episodic-like memory abilities across taxa.

## Data Availability

This article has no additional data.
